# A Case of Generalized, Unrelenting Sweating Resulting in Social Isolation for Over Two Decades

**DOI:** 10.7759/cureus.42339

**Published:** 2023-07-23

**Authors:** Anil Harrison, Caroline Tashdjian, Mayank Rampal

**Affiliations:** 1 Internal Medicine, University of Central Florida/HCA Florida West Hospital, Pensacola, USA; 2 Internal Medicine, Dignity Health St. Joseph’s Medical Center, Stockton, USA

**Keywords:** primary hyperhidrosis, sweating, catecholamines excess, neuroendocrine tumor, social isolation, sympathetic block, hyperhidrosis, pheochromocytoma

## Abstract

Pheochromocytoma, a rare neuroendocrine tumor, affects less than 1 in 100,000 people per year. Individuals with pheochromocytoma usually present with headache, diaphoresis, and tachycardia; however, diaphoresis occurs in less than half of the patients. Diaphoresis or chronic persistent hyperhidrosis can significantly impact patients’ lives, leading to depression, anxiety, and social isolation, as in our case. We report a patient with chronic persistent sweating for over two decades as the predominant manifestation of pheochromocytoma and sympathetic overdrive, affecting her mental and social well-being. Importantly, we would like to demonstrate the significance of long-standing sweating, which can impact an individual’s mental well-being and social life. Incorporating the Hyperhidrosis Disease Severity Measure-Axillary (HDSM-Ax) in the evaluation and management might be a relevant consideration. Pertinently, if medical measures and Botulinum toxin have failed to resolve excessive sweating, a thoracoscopic sympathetic block deserves consideration. Note that diaphoresis and hyperhidrosis, terms representing excessive sweating, will be used interchangeably.

## Introduction

Catecholamine-secreting neuroendocrine tumors of the chromaffin cells of the adrenal medulla, known as pheochromocytomas, are rare and have an incidence of about 0.6 cases per 100,000 person-years [1]. The diagnosis of pheochromocytoma is usually considered when the patient presents with headaches, profuse sweating, and an increase in heart rate, perceived as palpitations. Patients can present with a wide variety of clinical features, with episodic hypertension being the most reported, which is usually present in nearly 85% of all cases [2]. However, a combination of these three cardinal symptoms is present in less than one-fourth of patients diagnosed with pheochromocytoma [2], which is further confounded by anxiety and panic attack-related symptoms that are increasing in the population, making identification of these catecholamine-secreting tumors even more challenging for doctors. Interestingly, less than half of the patients with pheochromocytoma have diaphoresis/hyperhidrosis as the predominant symptom [3]. Pheochromocytoma can be diagnosed when both catecholamines (epinephrine, norepinephrine, and dopamine) rise using either plasma-free metanephrines or urinary fractionated metanephrines levels, and anatomically adrenal tumors can be documented. In the biochemical test for catecholamines, fractionated plasma metanephrines (normetanephrine and metanephrine) have a specificity of 93% and sensitivity of 97% across 15 studies [4]. If the biochemical test is positive, then an imaging evaluation is needed to confirm the tumor location anatomically either with a computed tomography (contrast-enhanced) (CT) scan or with magnetic resonance imaging (MRI) (T2-weighted). After localizing the tumor, further total body imaging is debatable. Functional imaging (e.g., scintigraphy with ^123^I-labeled metaiodobenzylguanidine (MIBG) or positron-emission tomography (PET)-CT with ^68^Ga-labeled 1,4,7,10-tetraazacyclododecane-1,4,7,10-tetraacetic acid-octreotate (DOTATATE) or ^18^F-labeled l-dihydroxy phenylalanine (l-DOPA) is very effective in localizing pheochromocytomas [5]. Functional imaging can be used for searching whether multiple chromaffin tumors or metastatic diseases are present. We report a patient with chronic persistent sweating for over two decades affecting her mental and social well-being.

## Case presentation

A woman in her late thirties, with episodic hypertension for the last six years, presented with complaints of chronic persistent and generalized hyperhidrosis for over two decades. Hyperhidrosis was generalized, unrelenting, and lasted throughout the day and at night. Stress and exertion aggravated her significant sweating, while she had no relieving factors. She inquired about treatment options related to her unrelenting hyperhidrosis. Several over-the-counter, topical, and oral pharmacological agents and injectable botulinum toxin injections provided no relief for her severe hyperhidrosis. Subsequently, this patient became depressed, anxious, and a social recluse. 

Six years before her visit, she began experiencing intermittent episodes of anxiety, tremulousness, and palpitations. She was prescribed antidepressants, benzodiazepines, and beta-blockers, which did not alleviate her symptoms. Although our patient denied weight loss, skin rash, headaches, diarrhea, and chest or abdominal pain, she felt warm and flushed at times. The patient’s body mass index (BMI) was 28 kg/m^2^. While on metoprolol 50 mg/day for palpitations and episodic hypertension, her presenting vitals included a temperature of 98° F, a heart rate of 80 beats per minute (bpm), and a blood pressure of 130/80 mm Hg. A thorough review of symptoms was unremarkable. She denied using any recreational drug use, tobacco, or alcohol intake.

The initial presentation revealed to us significant generalized hyperhidrosis, involving the dorsal aspects of her hands and feet. The rest of her physical examination was essentially unremarkable. Her laboratory evaluation revealed normal values of complete blood count with differential, C-reactive protein, comprehensive metabolic profile, thyroid profile, and 24-hour urine for 5-hydroxy indole acetic acid (5HIAA) and 5-hydroxytryptamine (5HT). Chest X-ray and electrocardiogram, including a 48-hour Holter recording, were unremarkable. Several infectious etiologies such as tuberculosis, Lyme disease, brucellosis, ehrlichiosis, hepatitis B and C, HIV, and COVID-19 were ruled out. Two sets of blood cultures along with a urine culture were also unremarkable.

Catecholamine analysis was done during episodes of palpitation and excessive diaphoresis, which revealed mildly elevated plasma normetanephrine. Due to this discordance and strong clinical suspicion, we moved to repeat plasma and urinary catecholamine levels during such episodes. Subsequently, her urinary metanephrines and plasma normetanephrine were found to be elevated along with plasma and urinary epinephrine levels, as shown in Table 1.

**Table 1:** Biochemical analysis. PTH: parathyroid hormone

Test	Result	Range
PTH intact	36 pg/mL	
Normetanephrine 24-hour urine	456 µg/24 hr	131–612 µg/24hr
Normetanephrine plasma	169.3 pg/nL	0.0–110.1 pg/mL
Metanephrines plasma	23.3 pg/mL	0–88 pg/mL
Epinephrine plasma	149 pg/mL	supine <50 pg/mL; upright <95 pg/mL
Epinephrine 24-hour urine	29 mcg/24 hr	2–24 mcg/24 hr
Metanephrine 24-hour urine	519 mcg/24 hr	35–482mcg/24 hr

To document the discomfort caused by hyperhidrosis, the Hyperhidrosis Disease Severity Measure-Axillary: (HDSM-Ax) questionnaire [6] was utilized. It is an 11-item scale (0-4 scale per item; 0-44 total scale) and it represents a primary axillary hyperhidrosis symptom range of 0 (no sweating) to 44 (worst possible sweating). On this scale, the patient scored 42/44.

**Figure 1:**
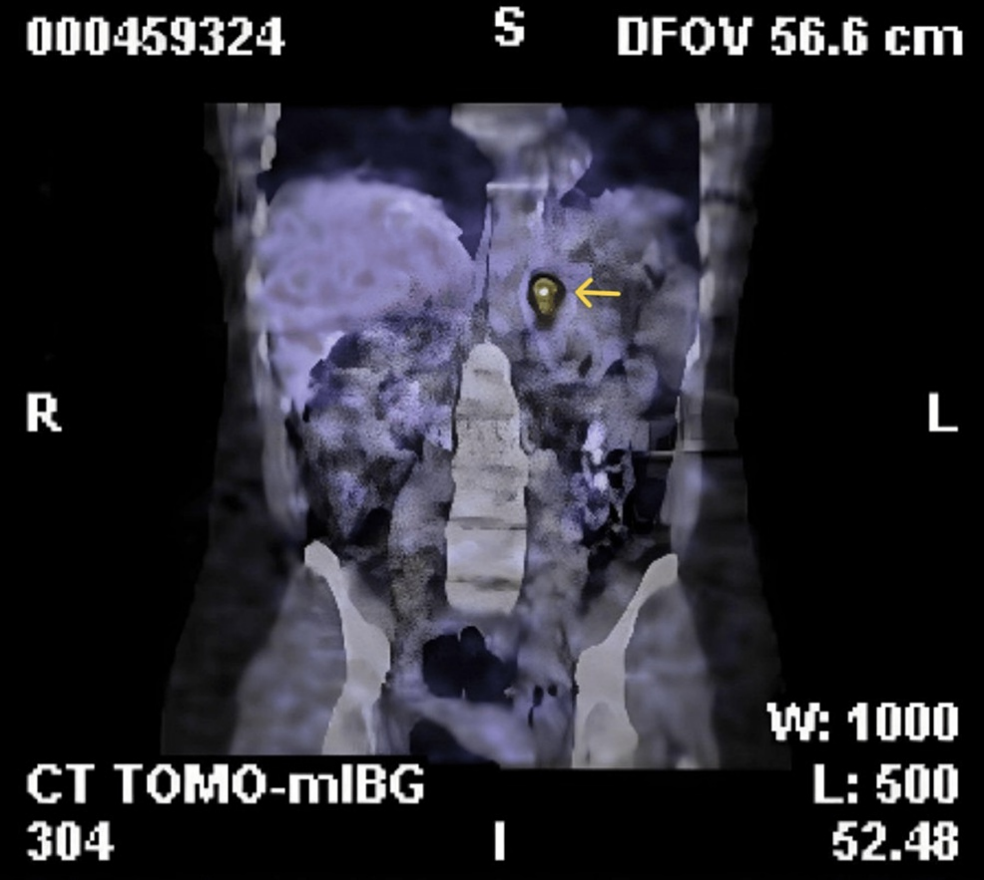
I-123 MIBG scan of the patient with an increased uptake in the left adrenal gland (yellow arrow) I-123 MIBG: 123I-labeled meta-iodobenzylguanidine

An MRI of her abdomen on T2-weighted demonstrated a 2.2 cm left adrenal nodule. An I-123-labeled MIBG scan, a functional imaging scan, demonstrated increased uptake in the left adrenal nodule with no other increased uptake suspicious for metastasis or other paragangliomas, suspicious for pheochromocytoma, shown in Figure 1.

Based on these cumulative findings, a diagnosis of pheochromocytoma was made and the patient was referred for surgery. Pre-operatively, the patient was started on phenoxybenzamine 10 mg at night for three days and then increased to 20 mg per day for one week, with the final dose increased to 40 mg per day along with labetalol in preparation for left adrenalectomy. 

After the procedure, pathology confirmed a completely excised pheochromocytoma. The patient underwent a battery of genetic testing for hereditary cancer syndromes, which were all negative. Post-op, after recovery, the patient had normal urinary and plasma catecholamines levels, was discharged, and was kept under regular follow-up. 

Six weeks later, the patient’s anxiety, palpitations, tremulousness, and blood pressure normalized, but her symptoms of excessive sweating only improved modestly. On utilizing the HDSM-Ax scale questionnaire, the patient's score was 34/44. After two decades of unsuccessful medical therapy, including Botox injections for her hyperhidrosis, the patient was given all available options and decided to undergo a video-assisted sympathetic block. Two weeks later, her HDSM-Ax scale questionnaire score dropped to 8/44, and she was satisfied with her treatment.

Currently, years after her treatment and multiple counseling sessions, our patient now is able to perform her job comfortably with good social interaction and live healthily. Also, her urinary and plasma catecholamine levels were found to be within the normal range.

## Discussion

Pheochromocytoma is a rare entity with a broad spectrum of possible presenting symptoms, making its diagnosis challenging for the treating team. The general practice to diagnose pheochromocytoma includes gathering evidence of raised catecholamine levels and anatomically identifying tumors.

Our patient, a female in her late thirties, presented with severe chronic persistent hyperhidrosis as the major symptom for over two decades, with intermittent palpitations, tremulousness, and anxiety, and episodic hypertension occurring just 6 years prior to her visit. Her symptoms began at age 12; however, unlike a majority of patients in that age group, she was not genetically predisposed to a pheochromocytoma.

When evaluating increased sweating, chronic hyperhidrosis or diaphoresis, infections (including tuberculous), catecholamine excess states, hyperthyroidism, insufficient production of adrenal hormones, lymphoproliferative and hematopoietic proliferative disorders need to be considered in the differential diagnosis. Physical exam, blood work and imaging studies ruled out all these possibilities, except for the possibility of a pheochromocytoma. 

Pheochromocytoma became the most likely diagnosis in the differential diagnosis, although we had to repeat plasma and urine biochemical studies for catecholamines due to our high suspicion. Furthermore, an MRI of the abdomen revealed a left adrenal nodule, which showed increased uptake on the I-123 MIBG scan, and no metastasis or other paragangliomas were seen, further raising our suspicion for pheochromocytoma.

The classic presentation of pheochromocytoma is due to the intermittent/episodic release of excess catecholamines (from the adrenal medulla) into circulation, resulting in palpitations, anxiety, diaphoresis, and headaches. This excess release can lead to increased cardiovascular morbidity and mortality [7], and events such as myocardial infarction, stroke, cardiomyopathy, peripheral vascular disease, and arrhythmias can occur, affecting up to 20% of those affected [8].

In this patient, excessive sweating/hyperhidrosis was the main symptom, occurring in less than 50% of patients with pheochromocytoma [2]. Hypertension, palpitations, and anxiety developed years later. The classic triad of tachycardia, headache, and diaphoresis occurs in only approximately 25% of such patients [2].

Sympathetic activity controls apocrine and eccrine sweat glands, although the eccrine sweat glands are also controlled by acetylcholine at the muscarinic receptors [9]. Peripheral vasoconstriction and heat dysregulation induced by increased sympathetic activity result in sweating. It is also postulated that excessive stimulus on the sympathetic fibers passing through the upper dorsal sympathetic ganglia, which accounts for neurons outside the adrenal gland, can result in sweating.

Our patient showed mild elevations in plasma normetanephrine and urine metanephrines, with diaphoresis being the only symptom for over two decades. This presentation of diaphoresis without other symptoms for many years has little evidence in the literature in the adolescent age group for pheochromocytoma. Later, the patient developed intermittent tremors, palpitations, anxiety, and hypertension, which was more consistent with pheochromocytoma and episodic release of excess catecholamines. Long-standing sweating can impact an individual's mental well-being and social life.

Incorporating the HDSM-Ax [6] in the evaluation and management should be a relevant consideration as it generates simple values ranging from no sweating to the worst sweating possible. Also, during follow-up with the patient, it helps quantify the symptom severity, which can guide clinicians to modify the treatment accordingly.

Surgical resection of the tumor is considered the cornerstone of the treatment. According to the 2014 guidelines issued for pheochromocytoma by the Endocrine Society, preoperative blockade of the adrenergic system was given [7]. Symptoms mostly resolve after the complete removal of the tumor, but in this patient, even after complete removal, she complained of persistent excessive sweating, which was documented to be 34/44 on the HDSM-Ax scale. This could be possible since even before surgery, catecholamine levels were not highly raised, so the patient was now suspected of having primary hyperhidrosis along with pheochromocytoma.

In a study by Noppen et al. on essential or primary hyperhidrosis, they proposed that primary hyperhidrosis is due to a poorly understood overstimulation of the sympathetic fibers passing through the upper D2 and D3 (dorsal) sympathetic ganglia [10]. Additionally, catecholamine levels may or may not be raised in such patients [10]. Pheochromocytoma could have been causing partial issues for the patient. In a study in adults by Lu et al. [11], patients with pheochromocytoma who had normal blood pressure had urine metanephrines at approximately 250 µg/24 hours, while in people with pheochromocytoma who had hypertension, the levels were more than three times this level. Tumor size is directly correlated with hormone level, and smaller tumors frequently secrete fewer catecholamines compared to larger tumors, which can have a wide variation in secretion potential. However, larger tumors secreted the highest hormone ratios [12], which can be correlated with our case. This suggests that the patient had both pheochromocytoma and primary hyperhidrosis.

The patient had a long history of finding no relief with medical management, including Botox injections. All available options were discussed with her, and she opted for a video-assisted sympathetic block. Two weeks later, the patient came for a follow-up, at which point she expressed her satisfaction with the treatment, and her HDSM-Ax score had now decreased to 8/44.

## Conclusions

Clinicians should be vigilant for pheochromocytoma as this disease is known to have increased cardiovascular morbidity and mortality. Pheochromocytoma can present with severe diaphoresis alone, with other symptoms such as palpitations and hypertension developing over time. When clinical suspicion is high, plasma and urine catecholamine levels should be repeated, especially if any discordant elevation is observed. Diaphoresis or chronic persistent hyperhidrosis can significantly impact patients' lives, leading to depression, anxiety, and social isolation. The HDSM-Ax scale is a measure of the severity of hyperhidrosis based on patient-reported symptoms, with a symptom range of 0 (no sweating) to 44 (severe sweating), which can be used in clinical research and practice to assess symptom severity and treatment response.

In the case of complete surgical resection of the pheochromocytoma tumor with persistent excessive sweating, primary hyperhidrosis should be considered. When various medical measures and multiple botulinum toxin injections do not provide relief to the patient, other measures, such as video-assisted sympathetic block, can be considered, which may result in near-complete resolution of the diaphoresis.

## References

[1] Berends AM, Buitenwerf E, de Krijger RR, Veeger NJ, van der Horst-Schrivers AN, Links TP, Kerstens MN (2018). Incidence of pheochromocytoma and sympathetic paraganglioma in the Netherlands: a nationwide study and systematic review. Eur J Intern Med.

[2] Geroula A, Deutschbein T, Langton K (2019). Pheochromocytoma and paraganglioma: clinical feature-based disease probability in relation to catecholamine biochemistry and reason for disease suspicion. Eur J Endocrinol.

[3] Manger WM, Gifford RJ (2012). Pheochromocytoma. https://books.google.co.in/books?hl=en&lr=&id=DSrjBwAAQBAJ&oi=fnd&pg=PR13&dq=Manger+WM,+Gifford+RW+Jr.+Pheochromocytoma.&ots=uze_TJwcO-&sig=tOrGx2lT77uWbnnWXFK9IYD7llE&redir_esc=y#v=onepage&q=Manger%20WM%2C%20Gifford%20RW%20Jr.%20Pheochromocytoma.&f=false.

[4] Lenders JW, Pacak K, Walther MM (2002). Biochemical diagnosis of pheochromocytoma: which test is best?. JAMA.

[5] Han S, Suh CH, Woo S, Kim YJ, Lee JJ (2019). Performance of (68)Ga-DOTA-conjugated somatostatin receptor-targeting peptide PET in detection of pheochromocytoma and paraganglioma: a systematic review and metaanalysis. J Nucl Med.

[6] Kirsch BM, Burke L, Hobart J, Angulo D, Walker PS (2018). The hyperhidrosis disease severity measure-axillary: conceptualization and development of item content. J Drugs Dermatol.

[7] Lenders JW, Duh QY, Eisenhofer G (2014). Pheochromocytoma and paraganglioma: an endocrine society clinical practice guideline. J Clin Endocrinol Metab.

[8] Zelinka T, Petrák O, Turková H (2012). High incidence of cardiovascular complications in pheochromocytoma. Horm Metab Res.

[9] Hu Y, Converse C, Lyons MC, Hsu WH (2018). Neural control of sweat secretion: a review. Br J Dermatol.

[10] Noppen M, Sevens C, Gerlo E, Vincken W (1997). Plasma catecholamine concentrations in essential hyperhidrosis and effects of thoracoscopic D2-D3 sympathicolysis. Eur J Clin Invest.

[11] Lu Y, Li P, Gan W (2016). Clinical and pathological characteristics of hypertensive and normotensive adrenal pheochromocytomas. Exp Clin Endocrinol Diabetes.

[12] Guerrero MA, Schreinemakers JM, Vriens MR (2009). Clinical spectrum of pheochromocytoma. J Am Coll Surg.

